# Effect of the Administration of a Nutraceutical Supplement in Racehorses with Lower Airway Inflammation

**DOI:** 10.3390/ani12182479

**Published:** 2022-09-19

**Authors:** Luca Stucchi, Chiara Maria Lo Feudo, Giovanni Stancari, Bianca Conturba, Francesco Ferrucci

**Affiliations:** 1 Veterinary Teaching Hospital, Department of Veterinary Medicine and Animal Sciences, Università degli Studi di Milano, 26900 Lodi, Italy; 2 Equine Sports Medicine Laboratory “Franco Tradati”, Department of Veterinary Medicine and Animal Sciences, Università degli Studi di Milano, 26900 Lodi, Italy

**Keywords:** equine asthma, racehorse, bronchoalveolar lavage, tracheal mucus, cough

## Abstract

**Simple Summary:**

The study of non-pharmacological products that could have a beneficial action on the respiratory system of the horse may have a great impact on the welfare and performance of equine athletes. As a matter of fact, young racehorses can be affected by a mild–moderate form of equine asthma (MEA), that can reach a prevalence of 80%, and pharmacological treatments during racing days are almost always forbidden. This study aimed to evaluate the efficacy of the administration of a feed supplement, composed of several nutraceutical herbs, in the treatment of lower airway inflammation. This product was administered to seven racehorses with MEA, while five horses were used as control. After 21 days, horses treated with the supplement showed a lower degree of clinical signs and lower mucus accumulation in the trachea, compared to controls. For this reason, the supplement was shown to be effective in controlling lower airway inflammation, and its use as an adjunctive treatment for MEA may deserve to be evaluated.

**Abstract:**

Mild–moderate equine asthma (MEA) is a chronic inflammatory disorder of the lower airways of the horse, characterized by tracheal mucus accumulation, cough and poor performance. The therapeutic approach is based on pharmacological treatment and environmental management. Moreover, the efficacy of the administration of antioxidant molecules has been reported. The aim of the present study was to evaluate the effect of the administration of a commercial nutraceutical supplement, composed of several herbal extracts, on lower airway inflammation in racehorses. Twelve Thoroughbreds affected by MEA were selected. All horses underwent a clinical examination with assignment of a clinical score, airway endoscopy and cytological examination of bronchoalveolar lavage fluid. In seven horses, the supplement was administered for 21 days in association with environmental changes, while in five horses only environmental changes were performed. All procedures were repeated at the end of the study. Data concerning the clinical score, the endoscopic scores and the cytology at the beginning and at the end of the study were statistically compared. Data showed a significant reduction (*p* < 0.0156) of the clinical score and a significant reduction (*p* < 0.0156) of the tracheal mucus score. The results showed the beneficial effect of the supplement on mild–moderate lower airway inflammation, probably due to its antioxidant activity.

## 1. Introduction

Young racehorses are often affected by an inflammatory disorder of the lower airways that is characterized by the presence of cough, mucus accumulation in the trachea and reduced athletic performance [1,2,3]. The diagnosis, that could be only presumptive on the basis of clinical symptoms and airway endoscopy, relies on cytological examination of the bronchoalveolar lavage fluid (BALf), where a pathological accumulation of neutrophils and/or eosinophils and/or mast cells can be detected [2,4]. This condition, that may affect up to 80% of Thoroughbred racehorses in training, has been recently classified as a mild or moderate form of equine asthma (MEA), due to its similarities with human asthma [5,6,7,8]. The treatment of this disease is based, similarly to human asthma, on the administration of bronchodilators and corticosteroids, both systemic and by inhalation, in association with changes in the environmental management, aimed to reduce the respirable dust [9,10,11].

Nevertheless, the presence of pharmacological molecules in the horse organism is forbidden during the racing day for most of the European Racing Association [12]. Therefore, the research on non-pharmacological molecules that may have beneficial effects on the respiratory system of the horse is of primary interest.

Recently, a complementary feed supplement has been introduced to the market, as a nutraceutical support for the respiratory system of the horse. It contains maltodextrin, calcium carbonate, *Arthrospira platensis* (12%) and fermented pineapple (5%), plus several herbal extracts and antioxidant substances. This supplement aims to reduce airway inflammation, primarily modulating the oxidative stress. In fact, it has been reported that oxidative status plays a major role in sustaining equine asthma [13,14,15]; in particular, neutrophils are responsible for the production of reactive oxidant species (ROS) and their accumulation in the lower airway can result in a high level of oxidative stress, leading to an increase in the inflammation [16,17]. Moreover, it has been demonstrated that the administration of antioxidant substances such as Omega-3 fatty acids, in association with environmental changes, was effective in reducing airway inflammation in horses affected by equine asthma [18].

The hypothesis for this work was that the nutraceutical substances contained in the supplement may have an effective anti-inflammatory action on the horses’ respiratory system. Thus, the aim of the study was to evaluate the efficacy of the administration of this supplement in the control of clinical symptoms, improvement of endoscopic findings and reduction of inflammatory cells in the lower airways of Thoroughbred racehorses in training affected by MEA.

## 2. Materials and Methods

### 2.1. Sample Selection

To perform the study, 12 client-owned Thoroughbred racehorses in training, referred to the Equine Unit of the Veterinary Teaching Hospital (University of Milan, Italy), were selected. Horses from three different training centers located in the North of Italy were enrolled; the study was performed at the stables where the horses usually lived, from May to September 2021. All horses were stabled in boxes bedded with straw, fed hay and concentrate on the basis of their nutritional requirements, and trained once daily. Horses with a history of decreased performance and symptoms consistent with MEA were included. At the beginning of the study (T0), all horses underwent a clinical examination with the attribution of an adapted clinical score: 1 point for the presence of cough, 1 point for the presence of nasal discharge, from 0 to 2 points for respiratory rate increase and from 0 to 3 points for the severity of the sounds at lung auscultation [19]. Then, all horses underwent a diagnostic protocol that consisted of upper and lower airway endoscopy, BALf collection and cytological examination of the BALf.

All procedures performed on the horses were approved by the University of Milan Animal Welfare Organization (Protocol Number OPBA_39_2021) and included informed owner consent.

### 2.2. Airway Endoscopy and Attribution of Endoscopic Scores

Horses were contained with a twitch and sedated with detomidine hydrochloride (0.01 mg/kg IV; Domosedan; Vetoquinol, Italy). A flexible videoendoscope (EC-530WL-P, Fujifilm, Tokyo, Japan) was passed through the left nostril, and the upper and lower tracts of the respiratory system were visualized and recorded. Then, the recordings were blind reviewed by an expert veterinarian (L.S.) who assigned a score from 0 to 4 for pharyngeal lymphoid hyperplasia (PLH) [20], from 0 to 5 for tracheal mucus accumulation [21] and from 0 to 4 for the edema of the tracheal bifurcation [22].

### 2.3. BALf Collection and Cytological Examination

During the airway endoscopy, a BALf sample was obtained. To perform the BALf, 60 mL of a 0.5% lidocaine hydrochloride solution was sprayed at the level of the tracheal bifurcation in order to inhibit the coughing reflex; then, the endoscope was passed into the bronchial tree until it was wedged firmly within a segmental bronchus. Here, a 300 mL pre-warmed sterile saline 0.9% was instilled, and the fluid was immediately aspirated. The BALf sample was stored in sterile ethylenediaminetetraacetic acid (EDTA) tubes and processed within 90 min. To perform the cytological examination, a few drops of pooled BALf were cytocentrifugated (Rotofix 32, Hettich Cyto System, Tuttlingen, Germany) at 26 *g* for 5 min. The slides were air dried, stained with May-Grünwald Giemsa and Perl’s Prussian blue, and observed under a light microscope at 400× and 1000× for 400-cell leukocyte differential counting [23]. In order to be included in the study, the cytological examination of BALf had to be consistent with MEA, presenting a percentage of neutrophils > 5%, and/or mast cells > 2% and/or eosinophils > 1% [24].

### 2.4. Treatment

Once the diagnosis of MEA was made, horses were divided casually by coin-flip into two groups:

- The Treatment group, composed of 7 horses. Horses in this group underwent changes in the management aimed to reduce respirable dust, i.e., bedding with wood shaving and feeding wet hay [25]. Moreover, they were administered with the supplement (BURAN Candioli, Acel Pharma s.r.l., Beinasco, Italy) at the dosage recommended by the company (35 gr/daily, orally) for 21 days. The composition of the supplement is displayed in Table 1.
- The Control group, composed of 5 horses, that underwent the same environmental changes of the Treatment group, but did not receive the nutritional supplement.

No other changes of stabling, feeding or training were performed during the study. After 21 days (T1), all horses underwent the same clinical protocol performed at T0, including clinical examination and attribution of the clinical score, airway endoscopy with attribution of endoscopic scores, and BALf cytology. The operator assigning scores (L.S.) remained blinded to the group distribution.

### 2.5. Statistical Analysis

Data were reported in an electronic sheet and analyzed using a commercially available statistical software package (Prism Graphpad 9.1.0 for MacOs; San Diego, CA, USA). Data distribution was evaluated for normality using a Shapiro–Wilk test. The values of age, the clinical score, the endoscopic scores (PLH, tracheal mucus and edema of the tracheal bifurcation), and the results of the differential cell count of the BALf at T0 were compared between groups by means of unpaired t-test or Mann–Whitney test, on the basis of data distribution. The clinical score, the endoscopic scores, and the cytological results of the BALf obtained at T0 and T1 were compared within both groups by means of the paired t-test, if normally distributed, or Wilcoxon test, if not normally distributed. Statistical significance was set at *p* < 0.05.

## 3. Results

Data are reported as mean ± standard deviation if normally distributed, or as median and interquartile range (I.Q.R.) if not normally distributed.

### 3.1. Horses

The study population consisted of nine colts, two fillies and one gelding. The median age of the Treatment group was 3, I.Q.R. 2–3, while the age of the Control group was 3, I.Q.R. 2–4. The groups were age-matched as no statistical difference was found between the ages of the groups.

### 3.2. Clinical Score

**Figure 1:**
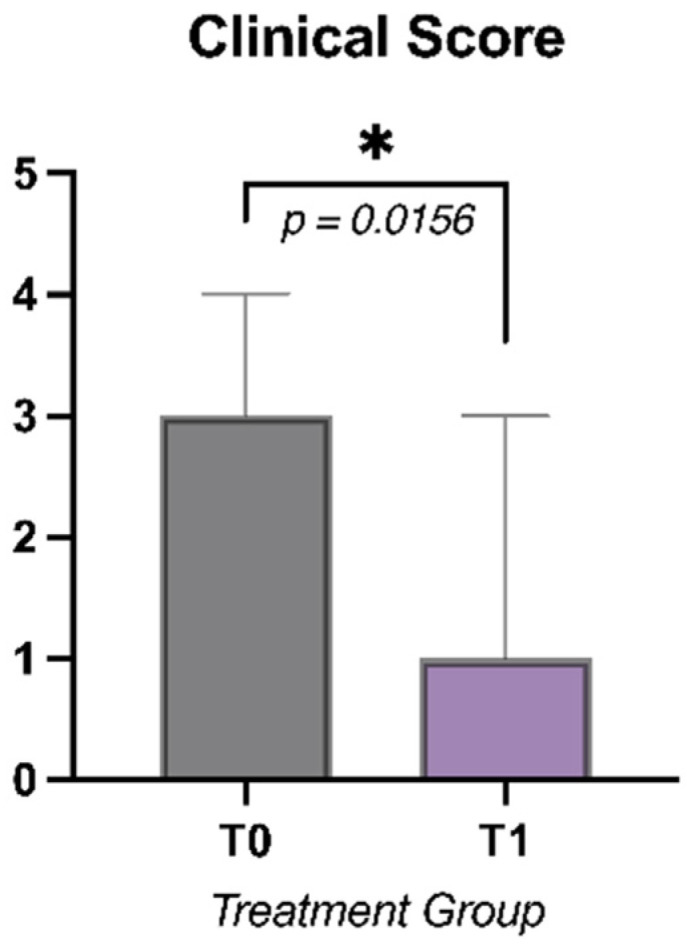
Clinical score at T0 and T1 in the Treatment group (* = *p*<0.05).

The results of clinical score in the two groups at T0 and T1 are reported in Table 2. A significant difference was detected (*p* = 0.0189) for the clinical score between the two groups at T0, with higher values in Treatment group. At T1, a significant difference (*p* = 0.0156) (Figure 1) was found in the Treatment group, while no significant difference was observed in the Control group.

### 3.3. Endoscopic Score

**Figure 2:**
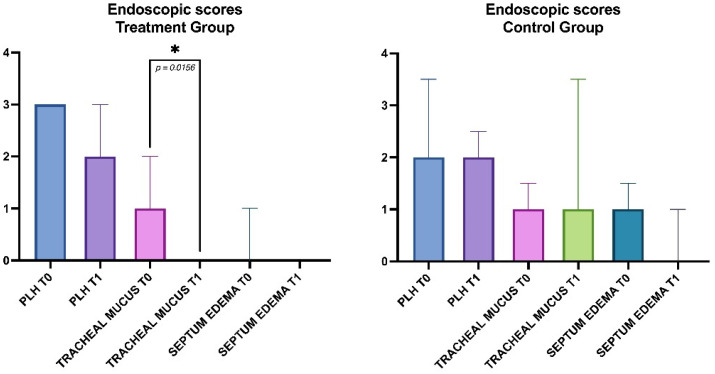
Results of the endoscopic scores at T0 and T1 in the two groups (* = *p*<0.05).

The results of the endoscopic score at T0 and T1 in the Treatment and Control groups are reported in Figure 2. No significant differences were observed between the two groups at T0 for all the endoscopic scores. At T1, a significant difference (*p* = 0.0156) in the tracheal mucus score was detected in the Treatment group, while no other differences were present in either group for the other scores.

### 3.4. Cytological Examination of the BALf

At T0, the total nucleated cell (TNC) numbers were 489 ± 31 for the Treatment group and 340 ± 168 for the Control Group, while at T1, they were 487 ± 24 and 332 ± 166, respectively. A significant difference in TNC was present (*p* = 0.028) between the two groups at T0. No significant differences were present between T0 and T1 in either group.

**Figure 3:**
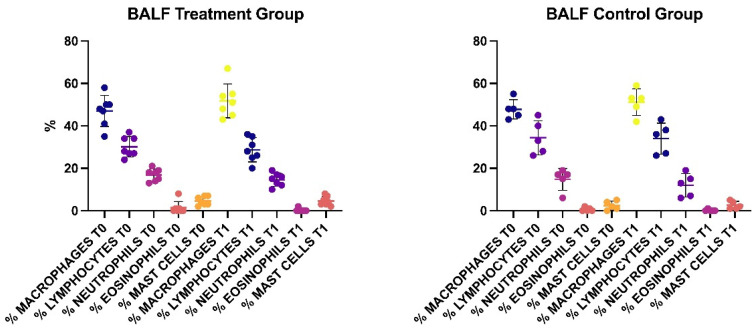
Results of the differential count of the inflammatory cells of the BALf in the two groups at T0 and T1.

The results of the differential count of the inflammatory cells of the BALf at T0 and T1 are reported in Figure 3. At T0, no significant differences were present between the two groups. At T1, no significant difference was found for all the cellular lines, either in the Treatment or in the Control group.

## 4. Discussion

The present study aimed to evaluate the effect of the administration of a nutraceutical supplement on mild lower airway inflammation of racehorses. The study of nonpharmacological products that could have a beneficial action on the respiratory system of the horse may have a big impact on the equestrian world, as equine asthma is widely distributed in the horse population and the use of medications during competitions is almost always forbidden.

The age of the horses involved in this study was very young, and this is in accordance with the definition of MEA [2]; in fact, MEA can occur at any age, but is more commonly reported in young horses [2]. As the percentage of neutrophils has been described to increase [26] and that of eosinophils to decrease with age [22], it was important that the groups were age matched. Concerning the sex, on the other hand, no data are available regarding the prevalence of MEA in a particular gender. It must be noticed that most of the horses in this study were colts, as is normal in a population of young Thoroughbreds in training.

At the beginning of the study, the endoscopic scores and the cytological profile were analogous in the Treatment group when compared to the Control group; moreover, both groups underwent the same management modifications during the treatment period. This choice was important, as it allowed us to standardize the study and to eliminate any possible bias that could have interfered with the results. We decided to use this cut-off of the BALf inflammatory cells for the diagnosis of MEA [24] as it can be considered the most used and accurate in racehorses [4,5].

The clinical score at T0 was higher in the Treatment group than the Control group: this could be due to the fact that MEA is a paucisymptomatic syndrome [2], and some clinical symptoms such as cough or abnormal respiratory sound at rest might not have been present. Nevertheless, it should be noticed that a lower score in the Control group at T0 could have influenced the statistical results regarding the reduction of clinical signs.

Statistical analysis showed that, at T1, there was a significant reduction of the clinical score and of the tracheal mucus score in the Treatment group, but not in the Control group. These results suggest that the administration of the supplement was effective in the reduction of clinical signs such as cough, nasal discharge or abnormal respiratory sound at lung auscultation, probably due to a decreased mucus production that was confirmed by tracheal endoscopy.

The mechanism underlying the efficacy of this product was not investigated in this work, but some explanations may be hypothesized by investigating the properties of some of its components. In fact, some papers showed the efficacy of *Allium sativum* in the mitigation of inflammation and mucus production in a mouse model of human asthma [27], and in the reduction of tracheal mucus in equine asthmatic patients [28]. Furthermore, *Thymus vulgaris* showed effects in the modulation of mucus hypersecretion in vitro [29], while *Hedera helix* seems to play an important role in reducing cough [30] and to have a mucolytic activity similar to acetylcysteine [31] in human patients with bronchitis. Finally, *Glycyrrhiza glabra* represents one of the most used and effective compounds for asthma in Chinese traditional medicine, and it was effective in reducing ROS production, bronchial inflammation, and mucus production in a murine model of human asthma [32,33].

Another explanation could be considered in the antioxidant effect of the supplement. In fact, it contains, in association with antioxidant vitamins C and E, a high percentage of *Arthrospira platensis* (12%), a herbal extract that has been proved to have an important scavenging activity against ROS [34], and has shown its protective effect on human bronchial tissue [35]. It is recognized that neutrophilic accumulation in the airways leads to an accumulation of ROS, resulting in an increase in mucus production and airway hyperreactivity [14]. In contrast, controlling the level of reactive species in the airways could induce a reduction of mucus accumulation in the trachea and a mitigation of cough, as observed in our study. Further studies regarding the oxidative status of the lung tissue after the administration of this supplement are needed to confirm this mechanism. Moreover, recently it has been demonstrated that the presence of high oxidative stress in the airways of the horse is one of the cause of the neutrophil corticosteroid insensitivity [17]; it could be hypothesized that the administration of products with antioxidant properties in association with standard therapies may be useful in the treatment of equine asthma.

Conversely, in our study we did not find any significant differences for PLH after the treatment period, either in the Treatment or in the Control group; this could be explained by the age of the population, as in young horses the presence of PLH is very common and not associated with severity of lower airway inflammation [22,36]. In the same manner, the absence of significant differences in the tracheal bifurcation edema score between T0 and T1 in both groups can be explained by the fact that this finding is more common in older horses affected by the severe form of equine asthma [22,37].

Concerning the cytological differential count of the BALf, no differences were found in either group after the treatment period; this results means that the cytological characteristic of the lower airway inflammation remained the same. However, it must be noticed that also conventional bronchodilator and corticosteroid therapy, even if from a clinical and mechanical point of view is rapidly effective, it determines a reduction of BALf neutrophilia only after 8 weeks of treatment [38]. It has also been proven that the best management modification to reduce lung inflammation is pasture [9], which unfortunately was not possible in our study.

## 5. Conclusions

In conclusion, the administration of the feed supplement, which is the subject of the present study, for 21 days, in association with some environmental changes, was effective in reducing clinical score and tracheal mucus score in a population of 12 Thoroughbred racehorses affected by MEA, but did not have any effect on BALf cytology. As MEA has a negative impact on the athletic capacity of the horses, for the future, it would be interesting to evaluate the effect of the administration of this product on performance parameters. Moreover, as exercise-induced pulmonary hemorrhage is another respiratory condition that is highly frequent in the racehorse population, it could be useful to also evaluate the effect of this nutraceutical supplement for this disease.

## Figures and Tables

**Table 1:** Herbal extracts and vitamins contained in the supplement.

Additives per kg	
*Allium sativum*	37.500 mg
*Glycyrrhiza glabra*	27.500 mg
*Thymus vulgaris*	18.000 mg
*Hedera helix*	7.500 mg
Vitamin C	50.000 mg
Vitamin E	30.000 mg

**Table 2:** Results of the clinical score at T0 and T1 in the Treatment and Control groups.

	T0				T1			
TREATMENT	Cough	Nasal	Respiratory	Lung	Cough	Nasal	Respiratory	Lung
		Discharge	Rate	Sound		Discharge	Rate	Sound
Horse 1	0	0	1	2	0	0	1	0
Horse 2	0	0	0	2	0	0	0	0
Horse 3	1	1	1	0	1	0	1	0
Horse 4	0	1	1	2	0	0	1	0
Horse 5	1	0	1	2	0	0	1	2
Horse 6	1	0	1	2	0	0	1	2
Horse 7	0	0	1	2	0	0	1	0
**CONTROL**								
Horse 1	0	0	0	0	0	0	0	0
Horse 2	0	0	0	1	0	0	0	0
Horse 3	0	0	0	2	0	0	0	0
Horse 4	0	0	1	0	1	0	0	2
Horse 5	0	0	1	2	1	0	1	2

## Data Availability

The data presented in this study are available on request from the corresponding authors.
